# *Yin Yang 1* is critical for mid-hindbrain neuroepithelium development and involved in cerebellar agenesis

**DOI:** 10.1186/s13041-020-00643-z

**Published:** 2020-07-23

**Authors:** Xiaonan Dong, Kin Ming Kwan

**Affiliations:** 1 School of Life Sciences, The Chinese University of Hong Kong, Hong Kong, Hong Kong SAR China; 2 Centre for Cell and Developmental Biology, The Chinese University of Hong Kong, Hong Kong, Hong Kong SAR China; 3 State Key Laboratory of Agrobiotechnology (CUHK), The Chinese University of Hong Kong, Hong Kong, Hong Kong SAR China

**Keywords:** *Yy1*, Cre-loxP, Cerebellar agenesis, Mid-hindbrain, Neuroepithelium, *Wnt1*, *p53*

## Abstract

The highly conserved and ubiquitously expressed transcription factor *Yin Yang 1* (*Yy1*), was named after its dual functions of both activating and repressing gene transcription. *Yy1* plays complex roles in various fundamental biological processes such as the cell cycle progression, cell proliferation, survival, and differentiation. Patients with dominant *Yy1* mutations suffer from central nervous system (CNS) developmental defects. However, the role of *Yy1* in mammalian CNS development remains to be fully elucidated. The isthmus organizer locates to the mid-hindbrain (MHB) boundary region and serves as the critical signaling center during midbrain and cerebellar early patterning. To study the function of *Yy1* in mesencephalon/ rhombomere 1 (mes/r1) neuroepithelium development, we utilized the tissue-specific *Cre-LoxP* system and generated a conditional knockout mouse line to inactivate *Yy1* in the MHB region. Mice with *Yy1* deletion in the mes/r1 region displayed cerebellar agenesis and dorsal midbrain hypoplasia. The *Yy1* deleted neuroepithelial cells underwent cell cycle arrest and apoptosis, with the concurrent changes of cell cycle regulatory genes expression, as well as activation of the p53 pathway. Moreover, we found that *Yy1* is involved in the transcriptional activation of *Wnt1* in neural stem cells. Thus, our work demonstrates the involvement of *Yy1* in cerebellar agenesis and the critical function of *Yy1* in mouse early MHB neuroepithelium maintenance and development.

## Introduction

Yin Yang 1 (YY1) is a ubiquitously expressed transcription factor which exerts multiple functions in various cellular events by activating or repressing gene transcription, modifying DNA conformation and controlling protein activity [1–4]. Numerous studies have suggested that YY1 regulates the activity of promoters and enhancers of genes that are implicated in the cell cycle, cell apoptosis, and cancer progression [3, 5]. The essential role of *Yy1* during embryogenesis was revealed by conventional deletion of *Yy1* in mouse embryos which led to peri-implantation lethality [6]. Intriguingly, a small subset of heterozygous *Yy1* depleted mouse embryos displayed exencephaly, asymmetric brain structure, and pseudo-ventricles, indicating a potential role of *Yy1* in mouse CNS patterning [6]. A dosage-dependent requirement of *Yy1* in late embryonic development was also reported by employing hypomorphic *Yy1* alleles containing mice [7].

On the other hand, human patients with the Gabriele-de Vries syndrome, which is caused by deletion or missense mutations of *Yy1*, suffer from neurologic symptoms including mental retardation, delayed psychomotor development, white matter abnormalities, delayed myelination and enlarged brain ventricles [8, 9]. The critical function of *Yy1* in oligodendrocyte differentiation has been reported through a conditional knockout mouse model [10]. Recently, a group of researchers uncovered that *Yy1* exerts a stage-dependent role by regulating metabolic pathways and protein synthesis during cerebral corticogenesis. In the mouse forebrain cortical neural progenitor cells (NPCs), *Yy1* controls cell proliferation and survival [11]. But the mechanism leading to neural developmental defects in other brain regions is still unclear.

The expression of *Yy1* can be found in the developing CNS of commonly used model organisms such as rodents and the *Xenopus* [12, 13]. Neurulation defects appeared when the homolog of YY1 (XYY1) in *Xenopus* is partially depleted [14]. XYY1 knockdown resulted in abnormal anterior-posterior patterning and reduction of head structures [13]. The gene expression profile of the XYY1 depleted embryos showed decreased expression of a group of patterning genes, including the homeobox genes, *Engrailed2*, *Otx2*, and *Krox20* [13]. Research focused on *Otx2* has revealed that its expression pattern relies on an enhancer containing a YY1 specific binding site. Disruption of YY1 binding resulted in the loss of *Otx2* expression in the anterior neuroepithelium [15]. But the mechanisms whereby *Yy1* affects most of the other genes’ expression during neural tube patterning remain to be elucidated. Moreover, the function of *Yy1* in mammalian early mid-hindbrain (MHB) neuroepithelium development is completely unknown.

The first step of vertebrate brain development is the subdivision of the neural plate. This regionalization step results in the formation of specific gene expression domains along the neural primordium [16]. The morphogenesis of the midbrain and the cerebellum is under precise control of the signaling center located at the boundary region, namely the isthmus organizer [17]. Members of several transcription factor families such as *Otx2, Gbx2, Engrailed (En)* and *Paired (Pax)*, have their restrictive expressing pattern and specific roles in the mid-hindbrain development [18]. The *Wnt* and *Fgf* families are the two major secreted factors at this phase [19]. Notably, *En1* is expressed starting from E8.5 in both the midbrain and rhombomere 1 regions.

We sought to uncover the function of *Yy1* in early embryonic neuroepithelial development. Here we demonstrated that the conditional knockout *Yy1* in the *En1*-expressed mesencephalon/ rhombomere 1 (mes/r1) neuroepithelium resulted in midbrain depletion and cerebellar agenesis. *Yy1*-deficient neuroepithelial cells (NECs) showed a reduction in proliferation and a dramatic increase in apoptosis due to the deregulated expression of some cell cycle regulators, especially the accumulation and stabilization of p53. We also observed the transcriptional activation of *Wnt1* by *Yy1* in NSCs which requires the binding of YY1 to the *Wnt1* promoter region. Our findings revealed the involvement of *Yy1* in cerebellar agenesis and a critical function of *Yy1* in mammalian MHB neuroepithelial cell survival and cell cycle progression.

## Results

### Conditional inactivation of *Yy1* in mouse mid-hindbrain boundary region

**Fig. 1:**
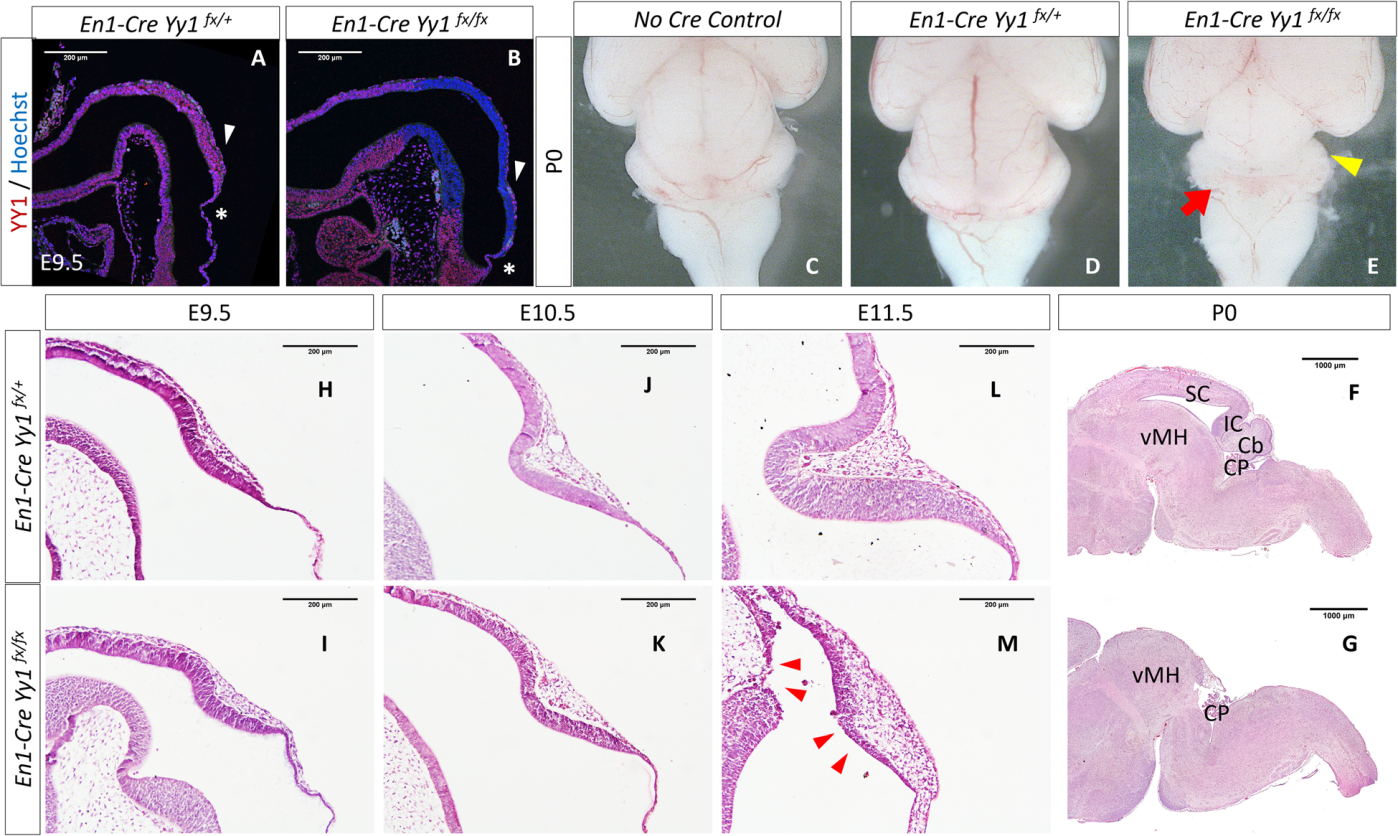
Brain morphological alteration of the En1-Cre-driven *Yy1* knockout mice. **a**, **b** Immunostaining with antibody against YY1 showing the complete inactivation of YY1 throughout the *Yy1* knockout mutant MHB neuroepithelium at E9.5. White arrowheads and asterisks pointing to the meninges and roof plate cells that still express YY1, respectively. **c-e** Gross morphology of no Cre control littermate (**c**), heterozygous (**d**), and homozygous mutant (**e**) at P0. The *En1*^*Cre/+*^*YY1*^*fx/+*^ mice showed no observable difference compared to the no Cre control littermates. Yellow arrowhead pointing to the truncated midbrain and missing cerebellum in *En1*^*Cre/+*^*; YY1*^*fx/fx*^ mutant. The red arrow pointing to the 4th ventricle choroid plexus. The figures were at the same magnification. **f**, **g** Histology of P0 control and mutant brain. **h-m** H&E staining of embryonic control and mutant littermates MHB region at E9.5, E10.5 and E11.5. Neuroepithelial tissue-loss could be observed in mutant at E11.5 (red arrowheads). Cb, cerebellum; CP, choroid plexus; IC, inferior colliculus; SC, superior colliculus; vMH, ventral mid-hindbrain

*Yy1* is expressed ubiquitously throughout embryonic development. To investigate the functional importance of *Yy1* in mammalian mid-hindbrain development, and to circumvent the embryonic lethality caused by conventional knockout, we employed the *Cre/LoxP* system to conditionally inactivate *Yy1* in mouse mes/r1 region around E8.5 by crossing the *En1*^*Cre/+*^ mice with *Yy1*^*flox/flox*^ carrying mice [20]. *En1*^*Cre/+*^*; Yy1*^*flox/+*^ mice were fertile and indistinguishable from their no-*Cre* littermates. We then backcrossed the *En1*^*Cre/+*^*; Yy1*^*flox/+*^ heterozygous with *Yy1*^*flox/flox*^ mice. The genotypes ratio of littermates delivered by the mating pairs followed the Mendelian ratio, however, homozygous *Yy1* inactivation driven by *En1-Cre* was perinatal lethal. All *En1*^*Cre/+*^*; Yy1*^*flox/flox*^ mice died within the first day after birth (P0). To confirm the inactivation of *Yy1* was thorough in the MHB neuroepithelium of homozygous conditional knockout mice, MHB regions from control and mutant littermates were dissected and sampled for qPCR and Western blot analysis. Due to the existence of non-NECs tissue, a background level of *Yy1* mRNA and protein could still be detected by these two methods, but we observed a persistent reduction of *Yy1* expression at E9.5 and E10.5 (Supplementary Figure S1A&B, Fig. 3e; For E10.5 qPCR, mutant 2^-ΔΔCq^ = 0.399 ± 0.026). We performed immunofluorescent staining of YY1 at E9.5 (Fig. 1a, b). Clearly, YY1 protein was completely undetectable in the mid-hindbrain neural tube of the *En1*^*Cre/+*^*; Yy1*^*flox/flox*^ mutant mice, but was still expressed in the meninges throughout the mes/r1 region (white arrowheads in Fig. 1a, b) and in the roof plate cells (white asterisks in Fig. 1a, b) that will develop into the 4th ventricle choroid plexus.

In the mutant mes/r1 neuroepithelia, we observed some sporadic YY1-positive cells (supplementary figure S1H). The percentage of YY1-positive NECs in *En1*^*Cre/+*^*; Yy1*^*flox/flox*^ mice mes/r1 slightly increased from E9.5 to E11, as the development proceeds (supplementary figure S1I). These small fractions of cells were only less than 2% of all. The great majority of mes/r1 cells (> 98%) completely lost the expression of *Yy1*, suggesting the knockout efficiency of En1-Cre in mes/r1 NECs was highly sufficient.

### Cerebella agenesis and midbrain hypoplasia in the MHB *Yy1* deleted mutants

The severe consequence of MHB *Yy1* ablation was first observed from the postmortem brains of P0 *En1*^*Cre/+*^*; Yy1*^*flox/flox*^ mice which lacked the superior colliculus, inferior colliculus, the cerebellum and its adjacent ventral hindbrain region (Fig. 1c-e). The 4th ventricular choroid plexus was normally hidden beneath the cerebellum but could be easily observed from the top-view of the mutant brain (Fig. 1e, red arrow). The remaining midbrain tegmentum fused with the posterior brain parts around the midline (Fig. 1f, g, supplementary Figure S1G). To trace the onset of the phenotype, we collected sampling pairs from E9.5 to E11.5 (Fig. 1h-m). The tissue-loss phenotype could be found at E11.5, indicated by the discontinuity of the mid-hindbrain neuroepithelium (red arrowheads in Fig. 1m). After *Yy1* inactivation, the remaining thin layer of NECs still expressed the neural stem cell marker SOX2 and SOX9 (supplementary Figure S1C-F) [21, 22], suggesting that inactivation of *Yy1* does not affect the neural stem cell identity of mes/r1 NECs.

### *Yy1* is required for proper cell cycle progression of the mid-hindbrain neuroepithelial cells

During neural tube development, the stem cells of neuroepithelial layer undergo rapid proliferation to accomplish the expansion of the neural tube. To test whether the mid-hindbrain defective phenotype of *Yy1* mutant was caused by the deficiency of cell proliferation, we adopted several cell cycle assays.

**Fig. 2:**
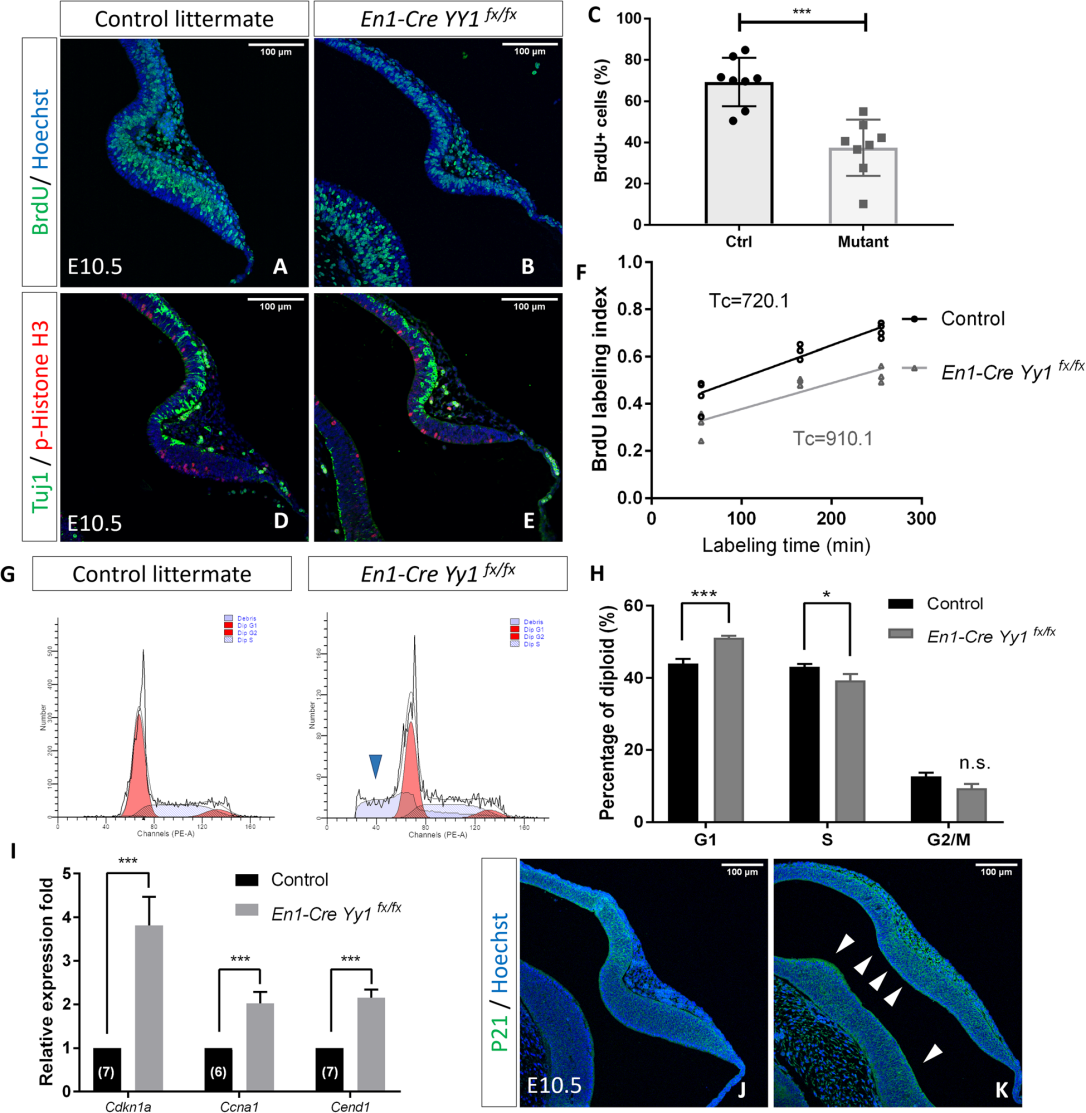
Knocking-out *Yy1* reduced proliferation of MHB NECs. **a**, **b** Immunofluorescent staining detecting 1-h labelled BrdU at E10.5. **c** Statistical analysis showing the percentage of BrdU-positive cells is reduced in mutant E10.5 MHB neuroepithelium. Two-tailed unpaired *t*-test, ***, *p* = 0.002*.* At least two sections counted from 4 pairs of 4 litters. **d**, **e** Staining of phospho-Histone H3 and Tuj1. **f** Cumulative BrdU-labeling experiments at E10.5 showing the lengthened cell-cycle time in mutants (control Tc = 720.1 min; mutant Tc = 910.1 min; correlation coefficients r2(control) = 0.9814, and r2(mutant) = 0.8861). **g** Representative flow cytometry results of control and mutant MHB dissociated cells stained with PI and analyzed by ModFit LT. Blue arrowhead pointing to the debris in mutant samples. **h** Statistical analysis of MHB cell cycle. Control, *n* = 10; Mutant, *n* = 7. Two-tailed unpaired *t*-test, *, *p* = 0.039; ***, *p* < 0.001. **i** Significant upregulated genes relative mRNA expression level. N numbers are shown in the bar of controls. Unpaired *t*-test, ***, *p* < 0.001. **j**, **k** Immunofluorescent imaging showing P21 expression increased in the mutant mes/r1 region

First, by detecting the proliferating cell nuclear antigen (PCNA) which is expressed at all stages of cell cycle, we did not observe a significant percentage change of cells within the cell cycle after *Yy1* ablation at E10.5 (supplementary Figure S2A & B). Then by utilizing the pulse-chase analysis, 1-h pulse of BrdU was administrated to pregnant mice at E10.5 (Fig. 2a & b). In contrast with the controls which underwent active proliferation (69.37 ± 4.138%, BrdU^+^ cells/ total cells counted, *n* = 8), the mutant MHB neuroepithelium reduced S-phase entry by almost half (37.44 ± 4.835%, BrdU^+^ cells/ total cells counted, n = 8) (Fig. 2c). To further characterize the proliferation properties of the *Yy1* deficient neuroepithelium, we analyzed the percentage of cells undergoing mitosis by detecting PhosphoHistone-H3. At E10.5, only a few cells were PhosphoHistone-H3-positive in wild-type mice MHB region (Fig. 2d). No significant reduction of metaphase cells was found in the MHB of *En1*^*Cre/+*^*; Yy1*^*flox/flox*^ mutants (Fig. 2e, Supplementary Figure S2C). We also observed no substantial change in the postmitotic cell ratio by detecting the β-III tubulin (TuJ1) in the MHB neuroepithelium (Fig. 2d & e, Supplementary Figure S2D).

The reduction of cells underwent DNA synthesis in the *Yy1*-ablated mes/r1 neuroepithelia lead us to investigate the cell cycle length of the NECs by accumulative BrdU labeling [23]. At E10.5, the cell cycle was lengthened in the *En1*^*Cre/+*^*; Yy1*^*flox/flox*^ NECs. The average cell-cycle time (Tc) of control NECs was 720.1 min, while the Tc of mutant NECs was 910.1 min (Fig. 2f).

To further obtain the cell cycle profile, mid-hindbrain neural tube region of E10.5 control and mutant pairs were dissociated into single cell suspension and fixed, followed by PI staining and examined by flow cytometry. Cell population was analyzed according to cell size and ploidy (Fig. 2g). Consistent with the other cell proliferation analysis data, we confirmed that the cells in the mutant MHB region have reduced S-phase entry (39.38 ± 1.74% for mutant vs. 43.19 ± 0.72% for control, *n* = 7), and the proportion of cells remaining at G1-phase increased significantly (51.22 ± 0.55% for mutant vs. 44.04 ± 1.26% for control, *n* = 7) (Fig. 2h, Supplementary Figure S2E).

To investigate the role and molecular mechanism of *Yy1* in NEC cell cycle progression, we analyzed whether inactivation of *Yy1* upset the major components of the cell cycle machinery, including the Cyclins, CDKs, and the cyclin-dependent kinase inhibitor. Among the factors we tested, we found no significant deregulation of the cell cycle regulators transcription, except *Cdkn1a (p21*^*cip1/waf1*^*, p21)*, *Ccna1* (*cyclin A1*), and *Cend1* (*Cell Cycle Exit And Neuronal Differentiation 1*) that were noticeably upregulated in the *Yy1* deficient neuroepithelium (Fig. 2i, Supplementary Figure S2F & G). At the protein level, both p21 and CEND1 exhibited more widespread expression patterns in the mutant mes/r1 neuroepithelia at E10.5 (Fig. 2j & k, Supplementary Figure 2H & I). The increased expression of these cell cycle regulators may contribute to the change of mutant NEC cell cycle length and progression. Taken together, our results suggest that *Yy1* regulates cell cycle length and G1-S progression in embryonic mes/r1 NECs.

### *Yy1* is required for the regulation of cleavage plane in mid-hindbrain neuroepithelial cells

**Fig. 3:**
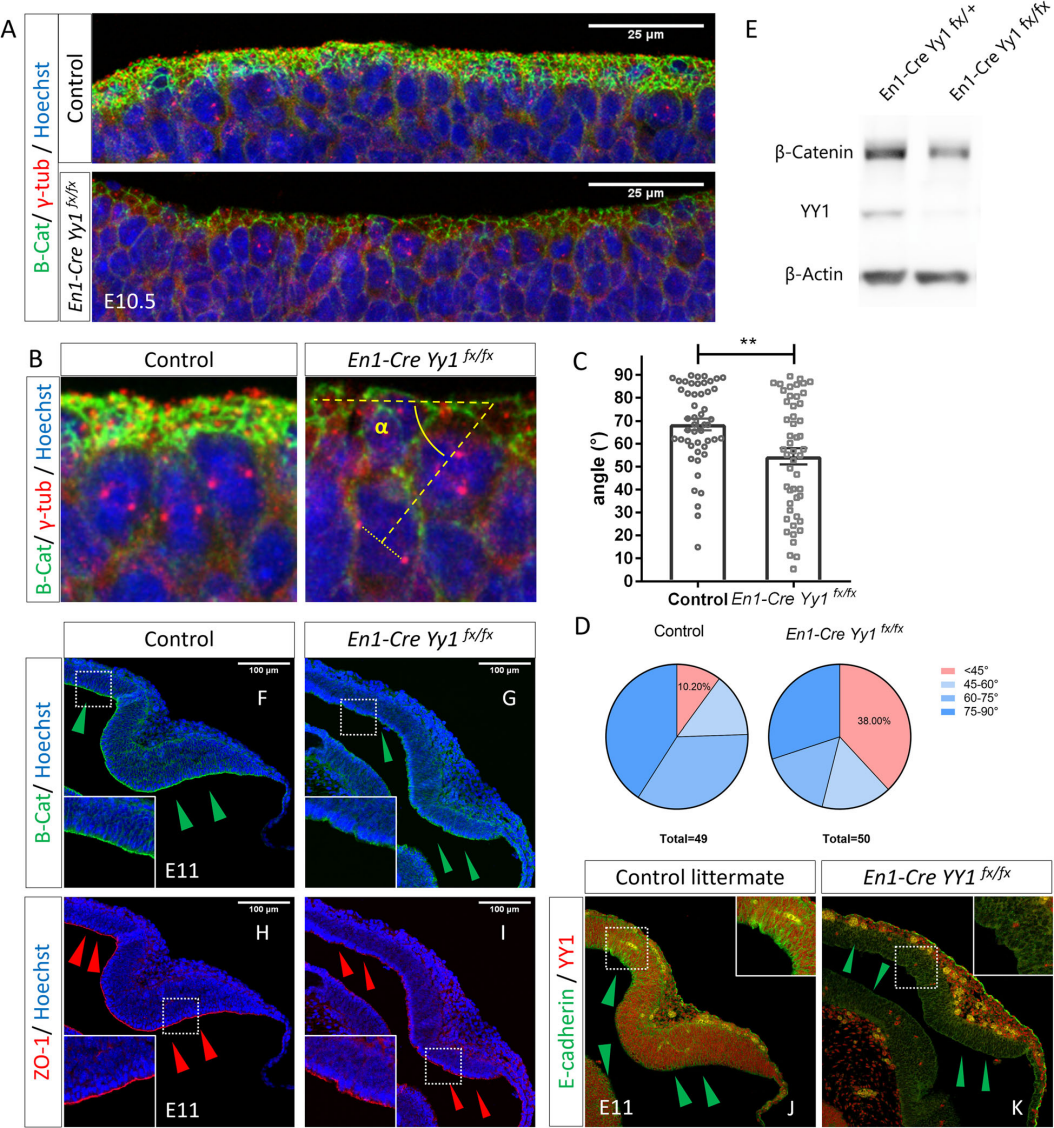
Inactivation of *Yy1* changed cell cleavage plane and decreased cell polarity proteins of MHB NECs. **a** Immunostaining of β-catenin and γ-tubulin in E10.5 MHB neuroepithelium showing loss-of-*Yy1* resulted in reduced β-catenin level and altered centrosome orientation. **b** Representative figure showing the measuring method of the mitotic spindle orientation angle. **c***Yy1-*ablated MHB NECs showed significant reduction in the mitotic spindle orientation angle. (the angle in the controls equals to 68.52 ± 2.544, *n* = 49, while in mutants is 54.58 ± 3.544, *n* = 50, **, *p* = 0.0019). **d** Mutant NECs showed significant increase in the percentage of cells undergoing asymmetric division. **e** Western blotting showing β-catenin level was reduced in *Yy1* KO mes/r1 region. **f-k** Immunostaining of cell polarity proteins β-catenin, ZO1 and E-cadherin showing at E11, deletion of *Yy1* in MHB NECs resulted in the partially destructed tissue integrity. Arrowheads showing reduced expression of the markers throughout the mes/r1 neuroepithelium

The expression of the cell cycle-related factor *Cend1*, also known as *BM88*, showed significant upregulation in the absence of *Yy1* expression (Fig. 2i, Supplementary Figure S2H & I)*,* same as previously reported [7, 24]. As its name suggests, *Cend1* controls the balance between cell cycle remaining and cell differentiation. The onset of neurogenesis process is switching the division plane from symmetric to asymmetric in the mitotic neuroepithelial cells, concomitant with the lengthening of cell cycle G1-phase [25–27]. In the previous section, we reported that the mutant NECs having a relatively longer cell cycle and G1-phase. So, we questioned if the mutant NECs switched to asymmetric cell division. The orientation of mitotic spindle is regulated by the position of centrosomes. The microtubule component γ-tubulin is commonly used as a marker for centrosome [28, 29]. To investigate if ablation of *Yy1* in mouse neuroepithelium affect the NEC division plane, immunostaining of γ-tubulin was performed to the E10.5 control and mutant MHB sagittal sections (Fig. 3a & b). At E10.5, the majority of wild-type MHB NECs underwent self-renewal (α > 45°, 89.80%, *n* = 49), with 40.82% of the cells have cleavage plane close to perpendicular (α = 75–90°, *n* = 49). A significant proportion of the *Yy1*-inactivated NECs altered their division plane to horizontal (α < 45°, 38.00%, *n* = 50), indicating loss-of-*Yy1* increased the asymmetric cell division of MHB NECs (Fig. 3b-d).

The cell-cycle progression and cell-cycle exit must be precisely regulated to guarantee the generation of the tissues with correct size, shape, and symmetry. NECs exhibit interkinetic nuclear migration and have the apical-basal polarity. The NECs are sensitive to the precise regulation of the mitotic spindle orientation during symmetrical proliferative divisions. The two daughter cells need to inherent both the apical and basal plasma membranes equally to ensure the cells to be attached at both the apical and basal surfaces of the neuroepithelium [30]. The combinatory alteration of cell cycle regulators forced the *Yy1*-inactivated NECs to switch their preference to asymmetrical cell division at E10.5. By long-term BrdU cell tracing, we found that the *Yy1*-inactivated mutant NECs successfully committed to neuronal differentiation fate (Supplementary Figure S2L & M). However, the outcome of increased asymmetric cell division of mutant NECs did not result in a significantly enriched differentiating cell population (Supplementary Figure S2D). It is possible that the altered mitotic spindle orientation may cause defects in cell attachment of the daughter cells, resulting in the cells end up with detachment from the neuroepithelium.

The neuroepithelium requires adherens junction for structural and functional maintenance. β-catenin, as a component of the complex, controls the adhesion and growth of the epithelial cells. Previous publication showed that β-catenin regulates the cleavage plane of midbrain NECs [31]. Reduced β-catenin level in midbrain caused misorientation of the mitotic spindle and premature differentiation [32]. In the *Yy1* ablated mouse mes/r1 neuroepithelium, we observed a looser structure of β-catenin network in the neuroepithelium apical surface (Fig. 3a). Western blot results also suggest the level of β-catenin throughout the MHB region of mutant is reduced when comparing to the control ones (Fig. 3e). When the development proceeds to E11, the integrity of apical β-catenin boundary is partially disrupted (Fig. 3f, g). In the area where cells bulge out, the cell polarity markers such as ZO-1, and E-Cadherin also showed alteration (Fig. 3h-k), which is consistent with the *En1-Cre; β-catenin*^*flox/flox*^ mouse phenotype [32]. These results suggest *Yy1* controls the cleavage plane of NECs and help to maintain the integrity of MHB neuroepithelium.

### Loss-of *Yy1* in MHB neuroepithelium resulted in p53 accumulation and elevated cell death

**Fig. 4:**
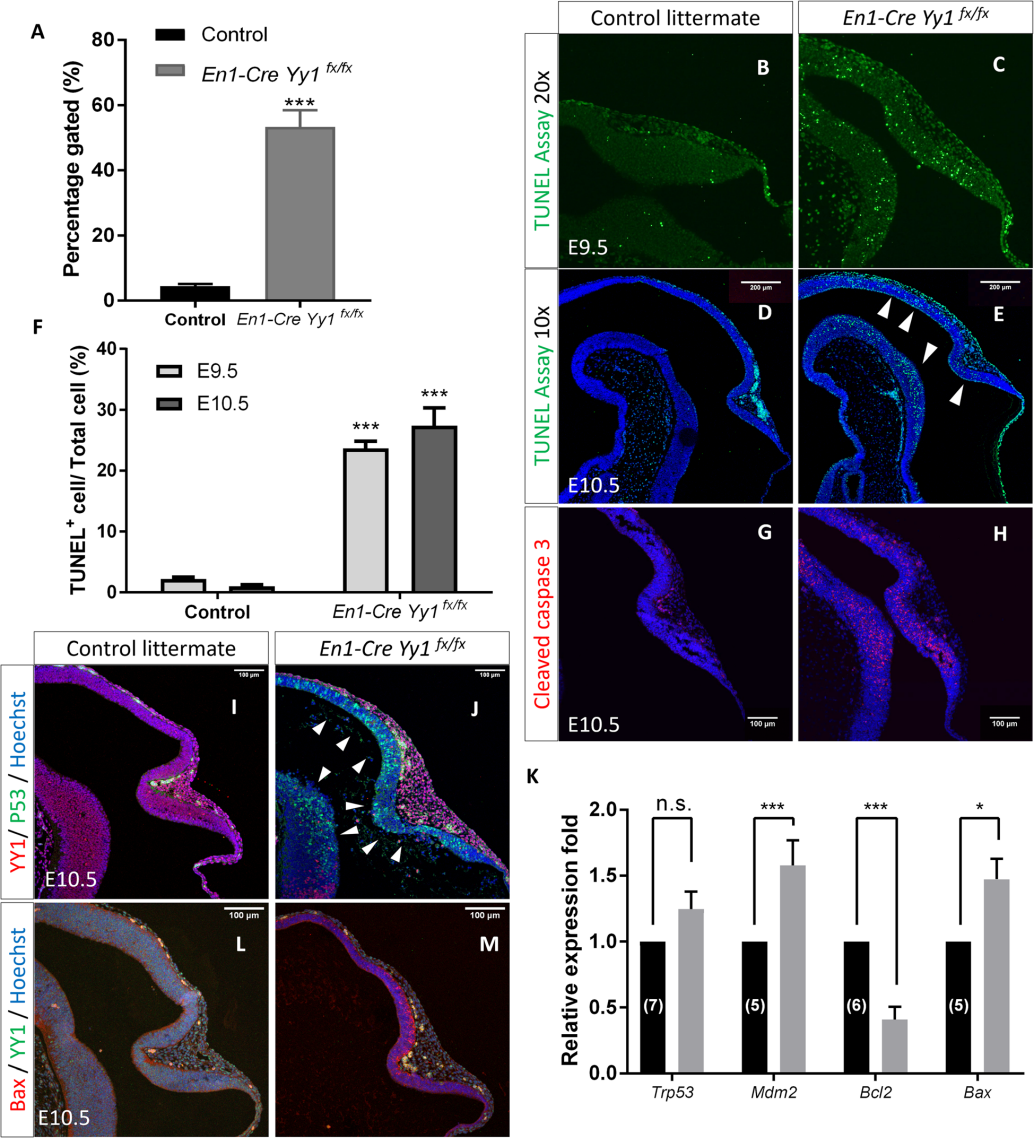
*Yy1* inactivation in MHB neuroepithelium caused apoptosis and p53 accumulation. **a** The percentage of debris in the gated dissociated mes/r1 cells from flow cytometry analysis. **b-e** TUNEL assay showing significant elevated cell apoptosis in the mutant MHB region at E9.5 (**b**, **c**) and E10.5 (**d**, **e**). **f** Statistical analysis showing dramatic increased apoptotic rate in the mutant MHB region. For E9.5, *N* = 4; For E10.5, *N* = 7. Unpaired two-tailed *t*-test. ***, *p* < 0.001. **g**, **h** Immunostaining using antibody against Cleaved Caspase 3 showing activation of Caspase cascade in mutant MHB neuroepithelium at E10.5. **i**, **j** Immunostaining showing nucleus accumulation of p53 (arrowheads) in the *Yy1*-deleted mes/r1 NECs at E10.5. **k** Relative expression level of *Trp53* and altered p53 pathway downstream genes. Unpaired *t*-test. ***, *p* < 0.001; *, *p* = 0.004. **l**, **m** Immunofluorescent staining showing the increased expression of the proapoptotic effector Bax in *Yy1*-ablated MHB neuroepithelium

We have demonstrated that lack of *Yy1* in NECs affected cell cycle progression, however, we do not think that this is the ruling mechanism contributing to the significant tissue loss in the mutant mid-hindbrain. As evident by the presence of a sharp increase in cell debris percentage, severe cell death might happen in the *Yy1*-deficient neuroepithelium (Fig. 4a, blue arrowhead in Fig. 2g). By using the TUNEL assay, a dramatically increased apoptotic rate was found in the *Yy1*-inactivated neuroepithelium at both E9.5 (1.597 ± 0.23% for control vs. 23.69 ± 1.15% for mutant, *n* = 4) (Fig. 4b, c, f) and E10.5 (1.027 ± 0.23% for control vs. 30.14 ± 2.63% for mutant, *n* = 7) (Fig. 4d-f). Similarly, immunostaining of the Cleaved Caspase 3, a major effector of the apoptotic cascade, labeled massive numbers of the *Yy1*-deleted NECs (Fig. 4g & h).

Based on the previous findings that *Yy1* may exert its anti-apoptotic function through regulating p53, we checked whether the protein level of p53 was altered in the *Yy1*-ablated MHB neuroepithelium [11, 33–36]. We detected a prominent accumulation of p53 in the cell nucleus of mutant mesencephalon and rhombomere 1 NECs (Fig. 4i-j), but no significant change at the mRNA level per se (Fig. 4k). However, the transcription of the p53-specific E3 ubiquitin ligase Mdm2 was significantly upregulated in the E9.5 mutant mes/r1 neuroepithelial tissue (Fig. 4k), indicating the mutant NECs aroused cellular mechanism in response to eliminate the accumulated p53. Gene expression profile analysis also indicates the profound deregulation of the intrinsic apoptotic pathway. There was decreased expression of pro-survival cell guardian *Bcl2* coupled with increased expression of pro-apoptotic effector *Bax* in the E9.5 mutant mes/r1 neuroepithelia (Fig. 4k-m). These data indicate a critical anti-apoptotic role of *Yy1* in MHB neuroepithelium, at least in part through regulating the p53 pathway.

### *Yy1* was required to maintain the expression level of *Wnt1* in mouse MHB

**Fig. 5:**
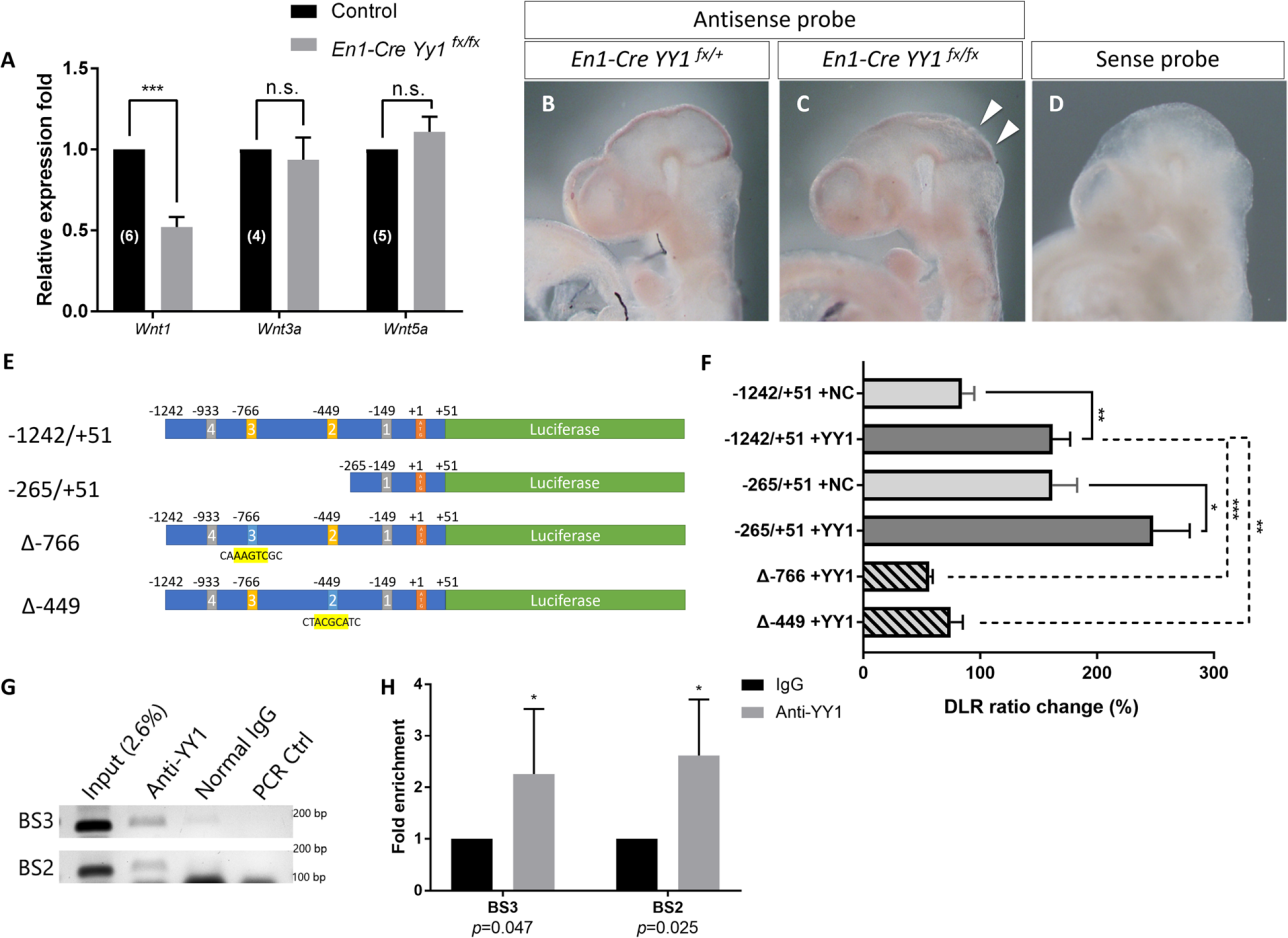
Inactivation of *Yy1* in mouse MHB reduced the expression of *Wnt1* by transcriptional deactivation. **a** Real-time qPCR results showing decreased expression of *Wnt1* but no other *Wnt* ligands in the *Yy1*-inactivated mes/r1 region at E9.5. N numbers for each gene tested are shown in the bars of controls. Unpaired *t*-test. ***, *p* < 0.001. **b-d** Whole-mount in situ hybridization using *Wnt1*-specific RNA probe showing reduced expression of *Wnt1* in mutant MHB region at E10.5. **e, f** Schematic illustration of *Wnt1* luciferase promoter analysis, with (**f**) showing the luciferase reporter assay results. Unpaired *t*-test, *n* = 6. For mutated binding sites, *n* = 4. *, *p* = 0.036; **, *p* < 0.01; ***, *p* < 0.001. **g** Representative results of gel electroporation using ChIP-qPCR products. **h** Fold enrichment of YY1-putative binding-site fragments immunoprecipitated with YY1. ChIP-qPCR results suggest YY1 binds to *Wnt1* promoter binding sites in E10.5 mouse mes/r1 cells. Each *p*-value is shown under the binding-site group number. Unpaired *t*-test, *N* = 4

Mid-hindbrain development is precisely controlled by an array of regional specific factors. By screening the potential MHB specification genes regulated by *Yy1*, we found that the expression of *Wnt1* was reduced by half in the mutant MHB notwithstanding the comparable level of other *Wnt* ligands (*Wnt3a* and *Wnt5a)* expressed within the MHB region at E9.5 (Fig. 5a). On the other hand, most of the crucial factors in MHB specification were unchanged, including *Otx2*, *Gbx2*, *En2*, *Pax5* and *Fgf8* (Supplementary Figure S3A & B). The reduced expression of *Wnt1* was further validated by whole-mount in situ hybridization (Fig. 5b-d). It is known that *Wnt1* is important for the maintenance and development of dorsal midbrain at early somite stages and of anterior hindbrain for the following later stages [37, 38]. The diminished expression of *Wnt1* as the consequence of *Yy1* ablation may affect cell proliferation and survival of the dorsal midbrain and rhombomere 1 neuroepithelial tissue at E9.5 and E10.5.

To understand the mechanism of *Yy1* regulating the expression of *Wnt1*, we analyzed the mouse *Wnt1* promoter and found four putative YY1-binding sites located within 1.2 kb, and one located just around the ATG of the first exon (− 2). After alignment with the human *Wnt1* promoter, two out of the four putative binding sites were shown to be conserved, we named them BS3 (− 766) and BS2 (− 449) according to their relative position to the ATG. We tested the promoter activity of these putative sites using the dual-luciferase reporter assay by co-transfecting the Flag-tagged-*Yy1* overexpression vector with luciferase reporter plasmids harboring *Wnt1* promoter fragments into the C17.2 neural stem cells. The YY1 expression level was on the average doubled 1-day after transfection as shown by the Western blot analysis (Supplementary Figure S3C). By overexpression of YY1, the luciferase activities were increased significantly for both the construct containing promoter region − 1242/+ 51 and the construct with truncated promoter fragment − 265/+ 51 (Fig. 5e, f). Moreover, the luciferase activity of the truncated promoter fragment − 265/+ 51 without both the two conserved putative YY1 binding sites was greater than the result of − 1242/+ 51, which suggests the existence of transcriptional inhibitory element within the promoter region − 1242/− 265. Significant reduction of luciferase activities were observed when we mutated either the conserved putative YY1 binding sites No.2 (Δ-449) or No.3 (Δ-766), even with the overexpression of YY1. This result suggests these YY1 putative binding sites may both be required at the same time for the transcriptional activation of the *Wnt1* promoter by YY1. When either No.2 or No.3 binding site is disabled, the transcriptional inhibitory element lies in the promoter region 1242/− 265 takes up the dominant place and inhibits the transcription activity of *Wnt1*.

To validate this YY1-*Wnt1*-promoter interaction in vivo, we performed ChIP-qPCR using E10.5 mouse MHB neuroepithelial tissue (Fig. 5g, h). We observed a 2-fold enrichment of the putative YY1-binding site fragments immunoprecipitated using YY1 antibodies when compared to the control group using normal IgG antibodies (For BS3, 2.26 ± 0.6316; For BS2, 2.619 ± 0.5432, *n* = 4.). These data suggest the presence of YY1 at the putative binding sites may help to activate *Wnt1* transcription in the embryonic MHB region at E10.5.

Taken together, our findings have elucidated the crucial function of *Yy1* in mammalian MHB neuroepithelial tissue maintenance, cell survival and cell cycle progression by regulating the degradation of p53 and the expression of *Wnt1*. Our study revealed that *Yy1* is indispensable during early mouse mid-hindbrain neural tube development.

## Discussion

### Diverse roles of *Yy1* in different cell types, cell differentiation stages and species

As a ubiquitously expressed transcription factor, YY1 plays complex roles during embryogenesis while the precise functions of YY1 differs in different cell types and developmental stages. For example, a previous study showed that YY1 participates in synergistic transcriptional activation of *Cdc6* by E2F2 and E2F3 with the facilitation of RYBP in embryo fibroblast cell [39]. However, we did not observe altered *Cdc6* transcription levels in the absence of YY1 in NECs (Supplementary Figure S2H) in our study. This result is similar to the conditional inactivation of *Yy1* in E11.5 mouse cortical NPCs. But when *Yy1* inactivation occurs at E15.5 late cortical radial glial cells, the expression of Cdc6 is significantly increased [11].

Human patients with *Yy1* haploinsufficient mutations display neurodevelopmental disorders [9]. A study on the forebrain suggests that *Yy1* dosage sensitivity in brain development differs from humans to mice [11]. Our MHB *Yy1* heterozygous mutant was also viable with a normal behavior which supports this suggestion. Moreover, although *Yy1* shows a highly conserved binding pattern through evolution, the detailed function of YY1 in regulating gene expression might differ between species. It has been reported that XYY1 levels influence the expression of *En2* in Xenopus [13]. But from our data, the *En2* expression was not significantly depleted in the *Yy1* knockout MHB region (Supplementary Figure S3B). These contradictory results suggest that there could be different regulatory mechanisms of *Yy1* in different systems.

### Regulatory function of *Yy1* in MHB NECs G1/S phase transition

The cyclins cooperate with their respective cyclin-dependent kinase (CDK) partners to regulate cell cycle progression. Within the mouse genome, there are two members in the A-type Cyclin family which exhibit distinctive expression patterns. CyclinA1 has a highly restricted expression pattern, the highest expression was found in the reproduction system, with relatively low levels of expression in the developing nervous system and limbs [40–43]. The *Ccna1* null mice displayed normal midbrain and cerebellar morphology [44]. In contrast, CyclinA2 is widely expressed in the cerebellum. Disrupted expression of *Ccna2* during cerebellum development reduced cerebellar volume and displayed cortical dyslamination [44]. It was suggested that in the developing cerebellum, CyclinA1 and A2 have no significant functional redundancy [44]. In the early cerebral cortical NPCs, *Yy1* deficiency did not influence the expression of Ccna1 or Ccna2 significantly. However, both CyclinA family members increased dramatically in late cortical NPCs [11]. In contrast to this result, we only observed upregulation of *Ccna1* but not *Ccna2*, however, the cause and effect of this deregulation remain to be investigated.

In eukaryotic cells the G1-S transition, as a critical event for cell cycle commitment, requires the E2Fs and their dimerization proteins. The E2F family of transcription factors bind to the promoters and control the transcription of cell cycle genes [45]. A previous study showed that *Yy1* binding sites were found in the E2F-regulated promoters such as *Cdc6*, *PCNA* and *Ccna*. With the help of the RYBP (Ring1- and YY1-binding protein) that YY1 interacts with the E2F2/ E2F3 family members and exert the combinatorial transcriptional activation function [39, 45]. From our results, the absence of *Yy1* did not cause significant downregulation of *PCNA*, *Cdc6* and *Ccna*, suggesting other YY1-independent intrinsic regulation or compensation may took place in the MHB NECs. We show that in the actively proliferating embryonic MHB NECs, *Yy1* is important for proper G1-S phase progression. The G1 phase of NECs was known to be lengthened concomitantly with the asymmetric division and neurogenesis progression [26]. The lengthening of G1 phase is necessary and sufficient to switch neural progenitors to differentiation, which is controlled by the Cdk4/CyclinD1 complex [46]. According to our results, besides *Ccna1*, the expression levels of *Cdks* and *Cyclins* were not changed significantly (Supplementary Figure S2F). We did not observe significant transcriptional inhibition of *Cdk4* or *Ccnd1*, which is similar to late stage inactivation of *Yy1* in cortical NPCs but not early-stage ones [11]. However, the cell cycle inhibitor *p21* was found to be upregulated, which may cause the Cdk–cyclin complex inhibition in the nucleus [47]. In addition, *Cend1* is expressed at a low level in neural progenitors but is upregulated in post-mitotic neurons [48]. Ablation of *Yy1* in the mouse MHB NECs reduced S-phase entry and resulted in upregulation of *Cend1*, which might be a mediator of *p21* overexpression and an inhibitor of CyclinD1 function [49]. However, after examination of the minimum promoter of *Cend1*, we did not observe any promising YY1-binding site [50]. This indicates that the regulation of *Yy1* on *Cend1* expression in NECs may not be through its conventional transcription inhibitory function. Taken together, although *Yy1-*ablated NSCs in both MHB and cerebral cortex displayed cell cycle arrest, our data suggest that the transcriptional responses affecting cell cycle machinery upon *Yy1* inactivation in early MHB NECs may differ from the later cortical NPCs, or between different brain regions. During embryonic brain development, the functional role for *Yy1* may vary in both a temporal and spatial manner.

### The regulation of p53 by YY1 may happen at multiple levels

The tumor suppressor *Trp53* is known to mediate growth arrest and apoptosis in response to various kinds of cellular stresses [51, 52]. The mRNA of *Trp53* can be found in the mouse neuroepithelium at E10.5 [53]. In 1997, two back-to-back papers reported p53 has a transactivation function in the developing mouse brain at around E10.5 [54, 55]. At this early developmental stage, *Trp53* may function as the guardian system against potential teratogenic insults. In the CNS, one previous study reported that ablation of *Yy1* in the oligodendrocyte progenitor neither affected the p53 level, nor caused any increased cell death [10]. However, a study in the cerebral cortical NSCs showed P53 accumulation in the embryonic *Yy1* conditional inactivated dorsal forebrain, and a double knockout of *p53* and *Yy1* rescued the reduced cortex size phenotype [11]. From our study, we showed that in the mouse MHB NECs, ablation of *Yy1* also caused accumulation of nuclear p53 and resulted in cell apoptosis. Taken together, we suggest that the activation of the p53 pathway is the critical mechanism causing early neural stem cell death in the circumstance of *Yy1* ablation, irrespective of whether the NSCs belong to forebrain or mid-hindbrain, but this p53 pathway may not be as critical in the more specified neuronal progenitor cells.

In the MHB NECs, this regulation might be achieved at three levels. First, as a transcription factor, p53 binds to downstream gene promoters in a sequence-specific manner [56, 57]. The consensus site of *Yy1* was found within a subset of *p53* binding elements of genes such as *p21* and *GADD45* that regulate DNA repair and cell cycle arrest [58]. The competition of YY1 binding to those sites inhibits the p53-activated transcription of the downstream genes. Without the perturbation of YY1, p53 signaling can be activated smoothly. Second, YY1 can disrupt the interaction of p53 and p300 thus blocking the p300-dependent acetylation and stabilization of p53 [34], and YY1 can facilitate the interaction between p53 and its ubiquitin ligase MDM2 [33]. In other words, YY1 controls p53 stability at the post-translational level. Third, it has been reported that *Cend1* overexpression triggers activation of p53 pathway [24]. Inactivation of *Yy1* caused upregulation of *Cend1*, and thus may contribute to the increased level of p53 protein to some extent.

### The possible contribution of *Wnt1* downregulation to the *Yy1*-cKO mid-hindbrain phenotype

The secretory glycoprotein WNT1 has been implicated as a key component that contributes to the isthmus organizer signaling activity. The expression of *Wnt1* in the MHB region is restricted to the mesencephalon and at the posterior limit of mesencephalon, but it is also crucial to the maintenance and development of the future cerebellum. Complete deletion of *Wnt1* resulted in cell loss of most of the *En1*-expressing cells within the mid-hindbrain region, which is comparable with our En1-Cre triggered *Yy1* conditional knockout mutant [59]. The *Wnt1*-null mutants displayed an absence of most of the midbrain and rostral metencephalon at E9.5 [37, 60]. The severe cerebellar defect caused by *Wnt1* mutations are generally attribute to aberrant organizer activity. On the other hand, *Wnt* is important to maintain neural stem cells self-renewal [61]. Misexpression of *Wnt1* in embryonic chicken brain resulted in enhanced cell proliferation and enlargement of brain regions, especially the mesencephalon [62]. Extending *Wnt1* expression to the *En1*-expressing region in mice resulted in increased cell proliferation only in the dorsal midbrain but not in the ventral midbrain [63]. The cell overproliferation follows a *Wnt1* dosage-dependent manner and is at least partially due to the shortening of the cell cycle length, which provides support for our hypothesis that the downregulation of *Wnt1* in *Yy1*-ablated MHB NECs contributes to cell cycle lengthening to some extent. More generally, overexpression of *Wnts* or persistent activated *Wnt* downstream effector in the CNS promotes cell cycle progression and inhibits cell-cycle exit [64]. Ectopic expression of *Wnt1* would promote G1-S phase transit and increased cell proliferation rate [65].

The transcriptional regulation of *Wnt1* is indeed achieved by the orchestration of large numbers of factors including transcription factors, epigenetic regulators, and non-coding RNAs. Our results suggest that YY1 takes parts in this regulatory network directly in vivo. The shortage of mesencephalic *Wnt1* may contribute to the lengthened cell cycle and increased in the abundance of cells which committed to asymmetric cell divisions during MHB neuroepithelium development.

## Methods

### Animals

The generation and genotyping methods of *En1*^*Cre/+*^ and *Yy1*^*flox/flox*^ C57BL/6 J mice were as previously described [7, 20, 66]. For genotyping, genomic DNA of embryonic and postnatal mice were obtained by digesting yolk sacs and toes, respectively. The mice were maintained on C57Bl/6 J background. *En1*^*Cre/+*^ bearing mice were mated with *Yy1*^*flox/flox*^ mice to generate heterozygous (*En1*^*Cre/+*^*; Yy1*^*flox/+*^) offspring. To generate *YY1* mid-hindbrain region conditional knockout mice (*En1*^*Cre/+*^*; Yy1*^*flox/flox*^*)*, *Yy1*^*flox/flox*^ females were crossed with *En1*^*Cre/+*^*; Yy1*^*flox/+*^ males. After we confirmed that the heterozygous conditional inactivation of *Yy1* did not cause any morphological defect, no-Cre and *En1*^*Cre/+*^*; Yy1*^*flox/+*^ littermate mice were used as the control. Control and mutant pairs from at least 3 litters were examined for each of the following assays.

Mice were raised in the animal house of the Chinese University of Hong Kong and kept in an artificial 10 h: 14 h, light: dark photoperiod with constant temperature and humidity. Animals were fed ad libitum with commercial rodent diet and distilled water. Females of each mating pairs were checked every morning, and the middays of the vaginal plug appeared were defined as embryonic day (E) 0.5. The day of birth was designated as postnatal day (P) 0. All animal procedures were handled in strict accordance to guidelines and approval given by the Animal Experimentation Ethics Committee of the Chinese University of Hong Kong.

### Histology and immunostaining

Embryos collected at the desired stages were fixed in 4% PFA in PBS at 4 °C overnight and then dehydrated through an ethanol series, embedded in paraffin and sagittal sectioned at 7 μm thickness. For histological analyses, sections were de-paraffinated and stained with hematoxylin and eosin. Images were captured with an Olympus microscope BX43 equipped with a DP72 CCD camera. For immunofluorescence staining, de-waxed sections were treated in boiled 10 mM sodium citrate buffer (pH 6.0) in a microwave oven for antigen retrieval. Sections were blocked at room temperature with BSA and horse serum, then incubated with primary antibodies at 4 °C overnight and corresponding secondary antibodies for 1 h at room temperature. Nuclei were counterstained with Hoechst 33342 (1:1000, Molecular Probes). The detail of antibodies used in this study are listed in Table 1.

**Table 1:** Antibodies used in this study

Antigen	Antibody name	Supplier	Cat. No.
β-actin	β-actin (C4): sc-47,778	santa cruz	sc-47,778
β-actin	β-actin antibody	Cell signaling	4967
β-Catenin	Purified Mouse anti-β Catenin	BD Transduction	610,153
γ-Tubulin	Anti-γ-Tubulin antibody produced in rabbit	sigma	T5192
Bax	Bax Antibody (B-9)	santa cruz	sc-7480
BrdU	Rat monoclonal [Bu1/75 (ICR1)] to BrdU	abcam	ab6326
CEND1	CEND1 (D6A6)	Cell signaling	8944
Cleaved Caspase-3	Cleaved Caspase-3 (Asp175) (5A1) Rabbit mAb	Cell signaling	9664
Cyclin D1	Cyclin D1 (92G2) Rabbit mAb	Cell signaling	2978
Cyclin D1	Anti-Cyclin D1 antibody [EPR2241]	Abcam	ab134175
E-cadherin	purified mouse anti-E-cadherin	BD Transduction	610,181
phospho-Histone H3	Anti-phospho-Histone H3 (Ser10), Mitosis Marker	Millipore	06–570
phospho-Histone H3	Anti-Histone H3 (phospho S10) antibody	abcam	ab14955
Ki67	Anti-Ki-67	Millipore	AB9260
p21	p21 (187)	Santa Cruz	sc-817
p53	Mouse anti-p53	Invitrogen	13–4100
PCNA	PCNA Antibody (PC10)	ThermoScientific	MA5–11358
PCNA	PCNA (D3H8P) XP Rabbit mAb	Cell signaling	13,110
Sox2	Sox2 (D9B8N) Rabbit mAb	Cell signaling	23,064
Sox9	Anti-Sox9	Millipore	AB5535
TUJ1	Purified anti-Tubulin beta 3 (TUBB3) antibody	Biolegend	801,202
YY1 for IF	anti-YY1 antibody	abcam	EPR4652
YY1 for IF	YY1 mouse monoclonal	Proteintech	66,281–1-Ig
YY1 for WB	anti-YY1 antibody	abcam	ab58068
ZO-1	Zo-1 / TJP1 Antibody	life tech	61–7300
Mus IgG	Alexa 488 Gt anti Mus IgG	invitrogen	A11029
Mus IgG	Alexa 568 Gt anti Mus IgG	invitrogen	A11031
Rat IgG	Alexa 647 Gt anti Rat IgG	Abcam	ab150159
Rb IgG	Alexa 488 Gt anti Rb IgG	invitrogen	A11034
Rb IgG	Alexa 568 Gt anti Rb IgG	invitrogen	A11036
Mus IgG	Goat anti- Mouse Horseradish peroxidase (HRP) conjugated affinity purifed secondary antibody	Millipore	AP181P
Rb IgG	Goat anti- Rabbit Horseradish peroxidase (HRP) conjugated affinity purifed secondary antibody	Millipore	AP187P

### Quantitative proliferation and apoptosis analysis

To label proliferating cells, fetuses were exposed to BrdU by an intraperitoneal injection of BrdU (10 mg/kg body weight) to the mother 1-h prior to sacrifice. The N numbers of this BrdU pulse labeling are shown in the figure, each dot represents the average BrdU^+^ cell percentage of one individual embryo. Samples were collected from 5 different litters. For the analysis of the cell-cycle exit, BrdU was injected to the mice 20 h before they were sacrificed. At least 3 sections from each animal were analyzed and averaged. For determination of the cell-cycle length, animals were injected with BrdU at E10.5 for 55 min, 165 min, and 255 min. At least 3 nonconsecutive sections per embryo were analyzed and averaged, each dot represents one embryo. For each time point, at least 3 control- mutant littermate pairs from 2 different litters were analyzed. The cell cycle length equals to 1/slope obtained from the linear regressions of the time series data of controls and mutants [23]. Samples were then prepared following the steps described in the immunostaining section.

TUNEL assays were performed on paraffin sections of E9.5 and E10.5 embryos by using In Situ Cell Death Detection Kit, Fluorescein (Sigma). Sections were counterstained with Hoechst and captured using a fluorescence microscope BX43 or confocal microscope SP8. The number of signal-positive cells and the total number of NECs were counted from the mes/r1 neuroepithelial region of at least 3 nonconsecutive sections. Four control-mutant littermate pairs from 3 different litters were used for samples.

### MHB cell dissociation and flow cytometric analysis

Control and mutant mouse mes/r1 tissues were dissected in Ca^2+^, Mg^2+^-free HBSS. The embryonic epidermal tissue was removed by micro-dissection. The neural epithelial tissue was treated with 0.05% trypsin/ EDTA at 37 °C for 10 min with gentle agitation. Digestion was then inhibited by adding HBSS containing 10% fetal bovine serum. After centrifugation and wash with PBS, dissociated cells were fixed by adding 1 ml − 20 °C 70% ethanol while vortexing. Permeabilization took place at − 20 °C overnight. After washing, the cells were treated with 100 μg/ ml RNase and 50 μg/ ml PI. The cell cycle profile was analyzed by using a flow cytometer BD FACSVerse with emission collected at 575–610 nm. Data of 10,000 events/ sample were analyzed using ModFit LT software. For *En1*^*Cre/+*^*; Yy1*^*fx/fx*^ mutants, *n* = 7 E10.5 individual embryos from 4 litters. For control, *n* = 10 individual embryos from the same litters of the mutants.

### Reverse-transcription and quantitative PCR

After removal of the epidermal tissue, the embryonic mid-hindbrain region neural tubes were dissected for RNA extraction. Total RNA was extracted with TRIzol (Invitrogen) following the manufacturer’s instructions. RNA samples used for reverse-transcription were measured by Nanodrop and adjusted to the equal final concentration. First strand cDNA was synthesized using M-MLV (Moloney murine leukemia virus) reverse transcriptase (Life Technologies) with oligo (dT)_20_ primers. Diluted cDNA samples were amplified by *Power* SYBR Green PCR Master Mix (Applied Biosystems) using specific primers listed in Table 2. Fluorescence was measured by Bio-Rad CFX96 Real-Time PCR Detection System at the end of each cycle. Each individual sample was assayed in duplicate and gene expression was normalized with *β-actin* expression. Gene expression was evaluated using 2 ^ (−*ΔΔCt*) method. The N numbers are shown in the control columns, represent the number of control and mutant littermate pairs tested for each gene.

**Table 2:** Primers for real-time qPCR

target gene name		primer sequence
βActin-qPCR	F	ACGCGCAGCCACTGT
	R	CTGACCCATTCCCACCATCA
bax-qPCR	F	TGCTAGCAAACTGGTGCTCA
	R	GCCTTCCCAGCCACCCT
Bcl2-qPCR	F	CAACATCGCCCTGTGGATGA
	R	AGGGTCTTCAGAGACAGCCA
Ccna1-qPCR	F	CTGACCGTTCCAACCACCAA
	R	TGCAGCAACCAAGGAAGGAA
Ccna2-qPCR	F	TTACCCGGAGCAAGAAAACC
	R	ACGTTCACTGGCTTGTCTTCTAA
Ccnb1-qPCR	F	AGTGACGTAGACGCAGATGAT
	R	GGTCTCCTGAAGCAGCCTAAA
Ccnd1-qPCR	F	GCGTACCCTGACACCAATCTC
	R	CTCCTCTTCGCACTTCTGCTC
Ccnd2-qPCR	F	GTACCCGCCGTCGATGATT
	R	CAGCAGCAGAGCTTCGATTT
Ccne1-qPCR	F	AGCACTTTCTGCAGCGTCAT
	R	TCAAAGAAGTCCTGTGCCAAGT
cdc6-qPCR	F	TCAGTCCCCGAAAACGTCTG
	R	TTCCACGTATGTGAGCGAGG
cdk1-qPCR	F	TGCAGGACTCCAGGCTGTAT
	R	AGGCCGAAATCAGCCAGTTT
cdk2-qPCR	F	GAGTCCCTGTCCGAACTTACA
	R	TCCTTGTGATGCAGCCACTTCTA
cdk4-qPCR	F	ATGTGGAGCGTTGGCTGTAT
	R	GGGCTCGGAAGGCAGAGATT
cdk6-qPCR	F	GCGTACCCACAGAAACCATAAAG
	R	CCGAGGTAAGGGCCATCTGAAA
cdkn1a-qPCR	F	CCTGGTGATGTCCGACCTG
	R	CCATGAGCGCATCGCAATC
Cend1-qPCR	F	CTCCTGAGCACTCCTCGGTAT
	R	GGTTTGGGGCTTGTGTGACT
Gbx2-qPCR	F	AGACGGCAAAGCCTTCTTG
	R	TCGGGTCATCTTCCACCTTT
Mdm2-qPCR	F	CCAGGCCAATGTGCAATACCAACA
	R	TGCGCTCCAACGGACTTTAACAAC
Otx2-qPCR	F	ATCTCCCTGAGAGCGGAACC
	R	CAGGGTCCTTGGTGGGTAGA
Pax5-qPCR	F	ATTACCCGACTCCTCGGACCAT
	R	TGATGGGCAAGTTCCACTATCC
Trp53-qPCR	F	ATCCTGGCTGTAGGTAGCGA
	R	ATCCGACTGTGACTCCTCCA
Wnt1-qPCR	F	GGTTTCTACTACGTTGCTACTGG
	R	GGAATCCGTCAACAGGTTCGT
Wnt3a-qPCR	F	CTCCTCTCGGATACCTCTTAGTG
	R	GCATGATCTCCACGTAGTTCCTG
Wnt5a-qPCR	F	CAACTGGCAGGACTTTCTCAA
	R	CATCTCCGATGCCGGAACT
Yy1-qPCR	F	GCAGAGTGTGGCAAAGCGTT
	R	CTGAGCAAACTTCTTATTACAACCG
Yy2-qPCR	F	TTGATGCCTGCAACAAGAAGT
	R	AGGCTTCAAAGGGACTCTCACT
Wnt1pro-bs2	F	CGGAGTCGCTGGCTAGAAC
	R	GCTGTCCTCTCGAAGTCCGT
Wnt1pro-bs3	F	TAGCCCACAGAGGCAAACTG
	R	CACTTCCCTCACCCAGGAAC
Wnt1pro-NC1	F	CCTCCCTTCCTTGTCCAACC
	R	CAATGCCTTTCGGGTCCTCT
Wnt1pro-NC2	F	TTCACTCCTGGGACCTCGAT
	R	GAGGAGGCTTTGGGAGACAC

### Cell culture and transfection

Mouse C17.2 neural stem cell line was a generous gift from Prof. PC Shaw, School of Life Sciences, CUHK. The cells were routinely cultured in Dulbecco’s Modified Eagle Medium (DMEM) with 10% fetal bovine serum (FBS, HyClone), 5% horse serum (HS) and 50 units/mL penicillin, and 50 μg/mL streptomycin (Gibco) at 37 °C with 5% CO_2_ in incubator. Culture medium was refreshed daily. Transfection was performed using jetPRIME reagent (Polyplus Transfection) when cells reached 60% confluence. The culture medium was then changed 4 h post-transfection.

### Dual luciferase reporter assay

Putative YY1 binding sites in the *Wnt1* promoter were predicted using online algorithms [67]. *Wnt1* promoter − 1378/+ 51 region was amplified from E9.5 mouse MHB gDNA and cloned into pGL3-basic (Promega). Luciferase expression plasmid containing truncated *Wnt1* promoter region − 1242/+ 51 and − 265/+ 51 were obtained by digesting the − 1378/+ 51 plasmid using KpnI and SmaI, respectively. For YY1 binding sites mutagenesis, mutations were introduced at the 5′ end of forward primers by substitution the template nucleotides. The primers were treated with polynucleotide kinase (PNK, NEB) prior to PCR. Template plasmids were eliminated by DpnI (NEB) digestion. The successful mutation of YY1 binding sites in the *Wnt1* promoter inserts were confirmed by full length sequencing. Primers used for mutagenesis were: for binding site No.2 forward: 5′-ACGCATCAGAATAGGGAAGAGAAGAG-3′, reverse: 5′-AGGATGAAAGTTGTGGTTCTAGCC-3′; for binding site No.3 forward: 5′-AAGTCGCTCTGTCTCCTTCTTTTCCTTC-3′, reverse: 5′-TGGAGCTGTGGTCAGGGATTCC-3′. The Dual luciferase reporter assay were performed according to the manufacturer’s instruction with pRL-SV40 as the internal control. Tagged mouse *Yy1* overexpression plasmid was purchased from Origene, pCMV6-entry empty vector was used as the negative control for co-transfection. Luminescence signals were read by CLARIOStar monochromator-based microplate reader.

### Chromatin-immunoprecipitation (ChIP)

The mes/r1 neuroepithelium ChIP was performed according to previous literatures [68, 69]. Generally, after removal of the embryonic skin, 4–5 wildtype C57/Bl6 E10.5 mouse mes/r1 neural tubes were dissected and pooled into ice-cold PBS. The tissues were minced with electoral homogenizer on ice. Samples were centrifuged at 2000 g for 5 min, resuspend in PBS with 1/10 (vol/vol) crosslinking buffer (11% PFA, 0.1 M NaCl, 1 mM EDTA, 0.5 mM EGTA and 50 mM HEPES, pH 7.6) and incubated at RT for 10 min with agitation. The crosslinking was quenched by adding 1/22 (vol/vol) 2.5 M glycine solution and incubated at RT for 5 min. Sample was centrifuged at 2000 g for 3 min, washed twice with PBS and resuspended with Lysis Buffer I (140 mM NaCl, 1 mM EDTA, 10% glycerol, 0.5% NP-40, 0.25% Triton X-100 and 50 mM HEPES, pH 7.6, with Complete Mini protease inhibitors) at RT for 10 min with agitation. Then sample was spun down at 2000 g for 10 min at 4 °C, resuspended in Lysis Buffer II (200 mM NaCl, 1 mM EDTA, 0.5 mM EGTA and 10 mM Tris, pH 7.6, with Complete Mini protease inhibitors) at RT for 10 min with agitation. After spinning down by centrifugation at 2000 g for 10 min at 4 °C, the sample was resuspended in Sonication Buffer (1 mM EDTA, 0.5 mM EGTA, 1% Triton-X100, 1% sodium deoxycholate and 10 mM Tris-HCl, pH 7.6, with Complete Mini protease inhibitors) and sonicated on ice for 20 times of 1 s-pulse at 50% power, 15 rounds with 1 min rest interval (Branson Sonifier 150). After sonication, the sample was centrifuged at 10,000 g for 10 min at 4 °C.

The chromatin solution was precleared by incubating with Protein G-Agarose for 1 h with agitation at 4 °C. The supernatant was collected by centrifugation at 1000 g for 5 min at 4 °C. The chromatin solution was then added together with respective antibodies and shook at 4 °C overnight. The antibody incubated chromatin solutions were then centrifuged at 12,000 g for 10 min at 4 °C, supernatants were collected.

The Dynabeads Protein G (Life Technologies) were used as the manufacture’s protocol. The Ab-chromatin supernatants were added to the pre-washed beads. Incubation took place in 4 °C with shaking for 2 h. The beads were collected using magnet and washed with 0.7 ml ice-cold RIPA buffer (0.7% deoxycholic acid, 1 mM EDTA, 0.5 M LiCl, 1% NP-40 and 50 mM HEPES, pH 7.6) for 5 times, incubated for 1 min on ice between each wash followed by washing with 0.7 ml ice-cold TE buffer (10 mM Tris-HCl, pH 8.0, and 0.1 mM EDTA) for 1 min on ice. The beads were collected and resuspended in Elution Buffer (50 mM Tris, pH 8.0, 10 mM EDTA and 1% SDS). Elution took place at 65 °C for 10 min and centrifuged at 14,000 g for 1 min. The supernatant was incubated at 65 °C overnight for reverse crosslinking.

On the next day, 1 mg/ml Proteinase K/ TE Buffer solution were added to the reaction and incubated at 55 °C for 2 h. DNA was extracted by phenol: chloroform. Pellets were collected and washed with 70% ethanol. After washing, pellet was air-dried and resuspended in milliQ water.

The purified DNA was then subjected to real-time PCR with primers listed in Table 2 to verify the presence of YY1 putative binding regions in the *Wnt1* promoter from the immunoprecipitated chromatin.

### Western blot analysis

MHB tissue or cells were lysed in protein lysis buffer (1% SDS, 10% glycerol, 5% β-mercaptoethanol and 125 mM Tris-HCl, pH 6.8) with proteinase inhibitor cocktail (cOmplete Mini, Roche). Samples were homogenized and centrifuged at 4 °C 12,000 rpm for 10 min. Supernatant was collected and boiled with loading buffer at 100 °C for 10 min. The denatured protein samples were then separated by 10% SDS-polyacrylamide gel electrophoresis. The proteins were trans-blotted to polyvinylidene difluoride membrane (FluoroTrans, PALL) by Bio-Rad Trans-Blot Turbo Transfer System for 15 min. The membranes were blocked with 5% non-fat dry milk in Tris-buffered saline with 0.1% Tween-20 (TBST) for 1 h with agitation at room temperature. The blocked membrane was incubated with 1:500 mouse anti-YY1 (Abcam, ab58068), 1:1000 rabbit anti-β-actin (Cell Signaling), 1:1000 mouse anti-β-actin (Cell Signaling), or 1:1000 rabbit anti-FLAG (Sigma) diluted in 2% ECL Prime Blocking Reagent/ TBST (GE Healthcare) at 4 °C overnight. After washing, the membrane was incubated with respective secondary antibodies conjugated to horseradish peroxidase (HRP) (1:2000, GE Healthcare) for 1 h at room temperature. The signal was detected using Western Blotting Substrate WESTSAVE Up (Abfrontier) by Chemidoc imaging system. For each experiment, at least three control and mutant littermate pairs from different litters were tested.

### Whole-mount in situ hybridization

Whole embryo RNA in situ hybridization were performed as previously described [70]. In brief, E9.5 mouse embryos were fixed in 4% PFA at 4 °C overnight and then dehydrated through methanol series. DIG-labeled RNA probes were synthesized using Roche transcription kit from linearized plasmid templates by following the manufacture’s protocol. Specimens were then rehydrated, digested with proteinase K and re-fixed in 4% PFA/ 0.2% glutaraldehyde at 4 °C for 20 min. After prehybridization, hybridization with probes took place overnight in hybridization buffer at 65–70 °C. Unbonded probes were washed away. Specimens were blocked at room temperature for 1 h and then incubated with pre-absorbed alkaline phosphatase-conjugated anti-DIG Fab fragments (Roche) overnight at 4 °C. After washes, alkaline phosphatase was detected using chromogenic conversion of NBT/ BCIP (both from Roche). Probes used for detecting *Fgf8* [70] was obtained or generated as cited.

Primers for cloning whole-mount in situ hybridization probe templates were *En2* forward: 5′-GAAGTCGACCGCTATCACTTCACGGTGGT-3′, reverse: 5′-ACGAGAATTCACTGGCCTTTTGTTCACGGT-3′. For *Wnt1,* forward: 5′-TATAGTCGACGGGCATCGTGAACATAGCCT-3′, reverse: 5′-CCGTGAATTCTTGGCGCATCTCAGAGAACA-3′. Successful cloning of DNA fragments was verified by sequencing. For all the experiments, at least three embryo littermate pairs were examined.

## Supplementary information

**Additional file 1: Figure S1.** (A) Relative mRNA level of *Yy1* and *Yy2* in E9.5 control and mutant mes/r1 tissue. N numbers are showed in the bars. Expression of mutants were compared with heterozygous littermates, ***, *p* < 0.001. (B) Protein expression level of YY1 was significantly reduced in the mutant dissected mes/r1 region. (C, D) Immunostaining using Sox2 antibody showing loss-of-*Yy1* did not influence the stem cell marker expression at E9.5. (E, F) Immunostaining of Sox9 showing the persistent expression of Sox9 at E10.5 in the *Yy1*-knockout cells. (G) H&E staining of the parasagittal sections of P0 control and mutant littermates. Red arrowheads pointing to the missing dorsal midbrain and cerebellum. (H) Immunostaining of YY1 showing very few random distributed YY1-positive cells in the E9.5, E10.5 and E11 mutant mes/r1 neuroepithelia. (I) Quantification of the YY1-positive NECs in mutant neuroepithelia.

**Additional file 2: Figure S2.** (A, B) Immunostaining of PCNA and YY1 in control and mutant mes/r1 region at E10.5. (C) Statistical quantification showing no significant change of M-phase cell percentage in E10.5 mutant neuroepithelia. Two-tailed unpaired t-test, *p* = 0.115. Samples were from 3 pairs of 2 litters. (D) Statistical analysis of Tuj1-positive cell percentage in E10.5 control and mutant mes/r1 neuroepithelia. Four control vsmutant littermate pairs from 3 different litters were analyzed. (E) Diagram showing the average proportion of cells in each phase of cell cycle. The average values are shown at the center of each bar. (F, G) Results of real-time qPCR cell cycle analysis. Among these factors, only the expression of *Ccna1* changed significantly, with ***, *p* < 0.001. (H, I) Representative sections of immunostaining of Cend1 at E10.5. (J, K) Representative sections of immunostaining of Cyclin D1 at E10.5. (L, M) Long-term BrdU cell tracing showing Tuj1^+^ BrdU^+^ cells in mutant mes/r1 neuroepithelium 20 h after labeling injection.

**Additional file 3: Figure S3.** (A) Real-time qPCR results showing no significant change of the regionalization markers *Otx2*, *Gbx2* and *Pax5* in E9.5 mutant mes/r1 region. (B) Whole-mount RNA in situ hybridization figures showing normal expression of *En2* and *Fgf8* in the *Yy1*-ablated mes/r1 region comparing to the control littermates. (C) Western blot analysis showing YY1 overexpression in C17.2 cells transfected with the pCMV6-MycFlag-*Yy1* plasmid.

## Data Availability

All data generated or analyzed during this study are included in this published article [and its supplementary information files].

## Abbreviations

BrdU: Bromodeoxyuridine
CDK: Cyclin-Dependent Kinase
ChIP: Chromatin-Immunoprecipitation
CNS: Central Nervous System
DMEM: Dulbecco’s Modified Eagle Medium
E: Embryonic Day
FBS: Fetal Bovine Serum
HBSS: Hank’s Balanced Salt Solution
HRP: Horseradish Peroxidase
HS: Horse Serum
Mes/r1: Mesencephalon and Rhombomere 1
MHB: Mid-Hindbrain
NECs: Neuroepithelial Cells
NPCs: Neural Progenitor Cells
NSCs: Neural Stem Cells
P: Postnatal Day
PBS: Phosphate-Buffered Saline
PCNA: Proliferating Cell Nuclear Antigen
PNK: Polynucleotide Kinase
TUNEL: Terminal Deoxynucleotidyl Transferase dUTP Nick End Labeling
