# Supplementary figures and images for: Yin Yang 1 is critical for mid-hindbrain neuroepithelium development and involved in cerebellar agenesis
Source: Mol Brain. 2020 Jul 23;13:104. doi: 10.1186/s13041-020-00643-z (PMC7376712; doi:10.1186/s13041-020-00643-z)

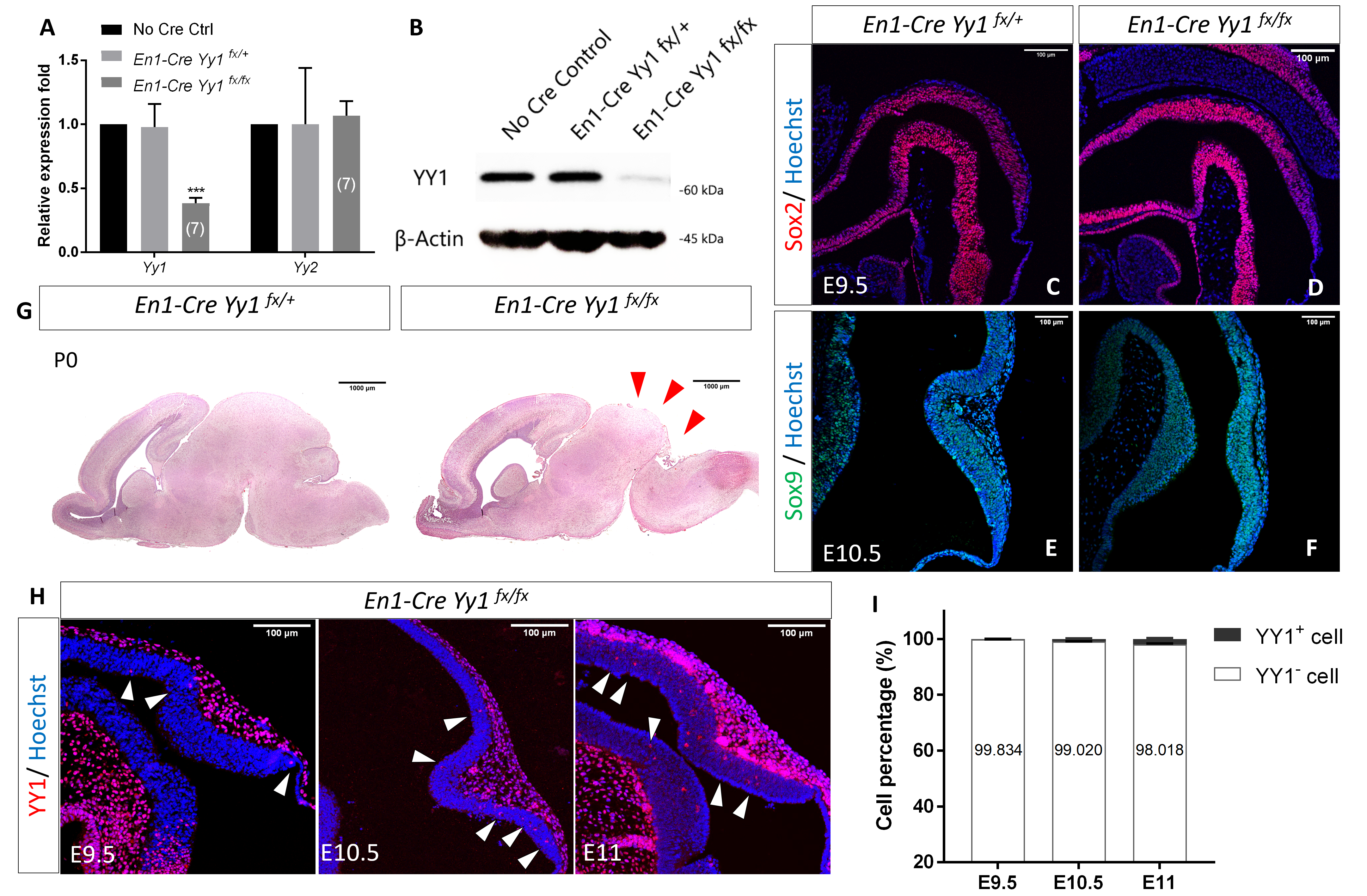

Supplement: Supplementary file 1 — Additional file 1: Figure S1. (A) Relative mRNA level of Yy1 and Yy2 in E9.5 control and mutant mes/r1 tissue. N numbers are showed in the bars. Expression of mutants were compared with heterozygous littermates, ***, p < 0.001. (B) Protein expression level of YY1 was significantly reduced in the mutant dissected mes/r1 region. (C, D) Immunostaining using Sox2 antibody showing loss-of-Yy1 did not influence the stem cell marker expression at E9.5. (E, F) Immunostaining of Sox9 showing the persistent expression of Sox9 at E10.5 in the Yy1-knockout cells. (G) H&E staining of the parasagittal sections of P0 control and mutant littermates. Red arrowheads pointing to the missing dorsal midbrain and cerebellum. (H) Immunostaining of YY1 showing very few random distributed YY1-positive cells in the E9.5, E10.5 and E11 mutant mes/r1 neuroepithelia. (I) Quantification of the YY1-positive NECs in mutant neuroepithelia. [file 13041_2020_643_MOESM1_ESM.tif]

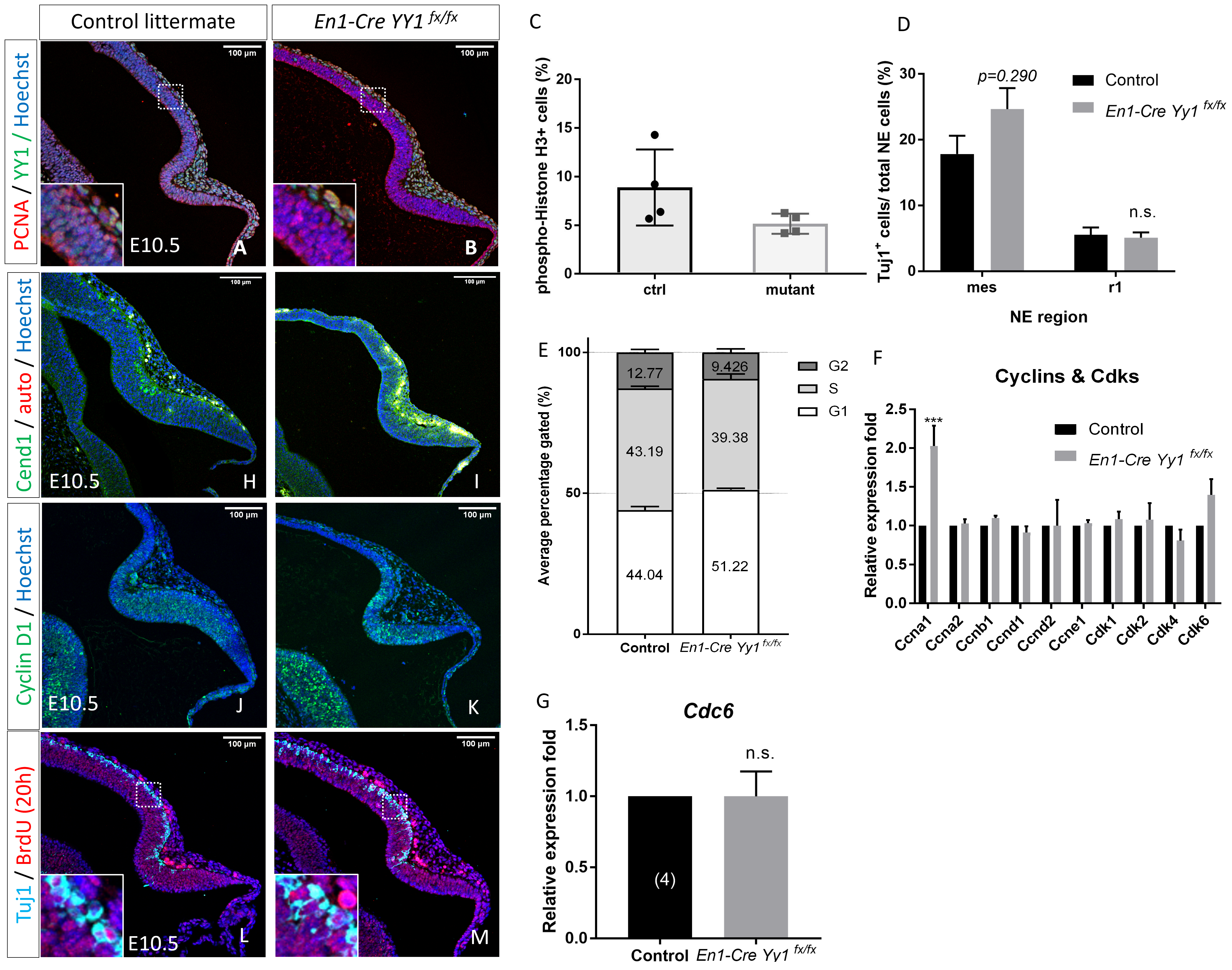

Supplement: Supplementary file 2 — Additional file 2: Figure S2. (A, B) Immunostaining of PCNA and YY1 in control and mutant mes/r1 region at E10.5. (C) Statistical quantification showing no significant change of M-phase cell percentage in E10.5 mutant neuroepithelia. Two-tailed unpaired t-test, p = 0.115. Samples were from 3 pairs of 2 litters. (D) Statistical analysis of Tuj1-positive cell percentage in E10.5 control and mutant mes/r1 neuroepithelia. Four control vsmutant littermate pairs from 3 different litters were analyzed. (E) Diagram showing the average proportion of cells in each phase of cell cycle. The average values are shown at the center of each bar. (F, G) Results of real-time qPCR cell cycle analysis. Among these factors, only the expression of Ccna1 changed significantly, with ***, p < 0.001. (H, I) Representative sections of immunostaining of Cend1 at E10.5. (J, K) Representative sections of immunostaining of Cyclin D1 at E10.5. (L, M) Long-term BrdU cell tracing showing Tuj1+ BrdU+ cells in mutant mes/r1 neuroepithelium 20 h after labeling injection. [file 13041_2020_643_MOESM2_ESM.tif]

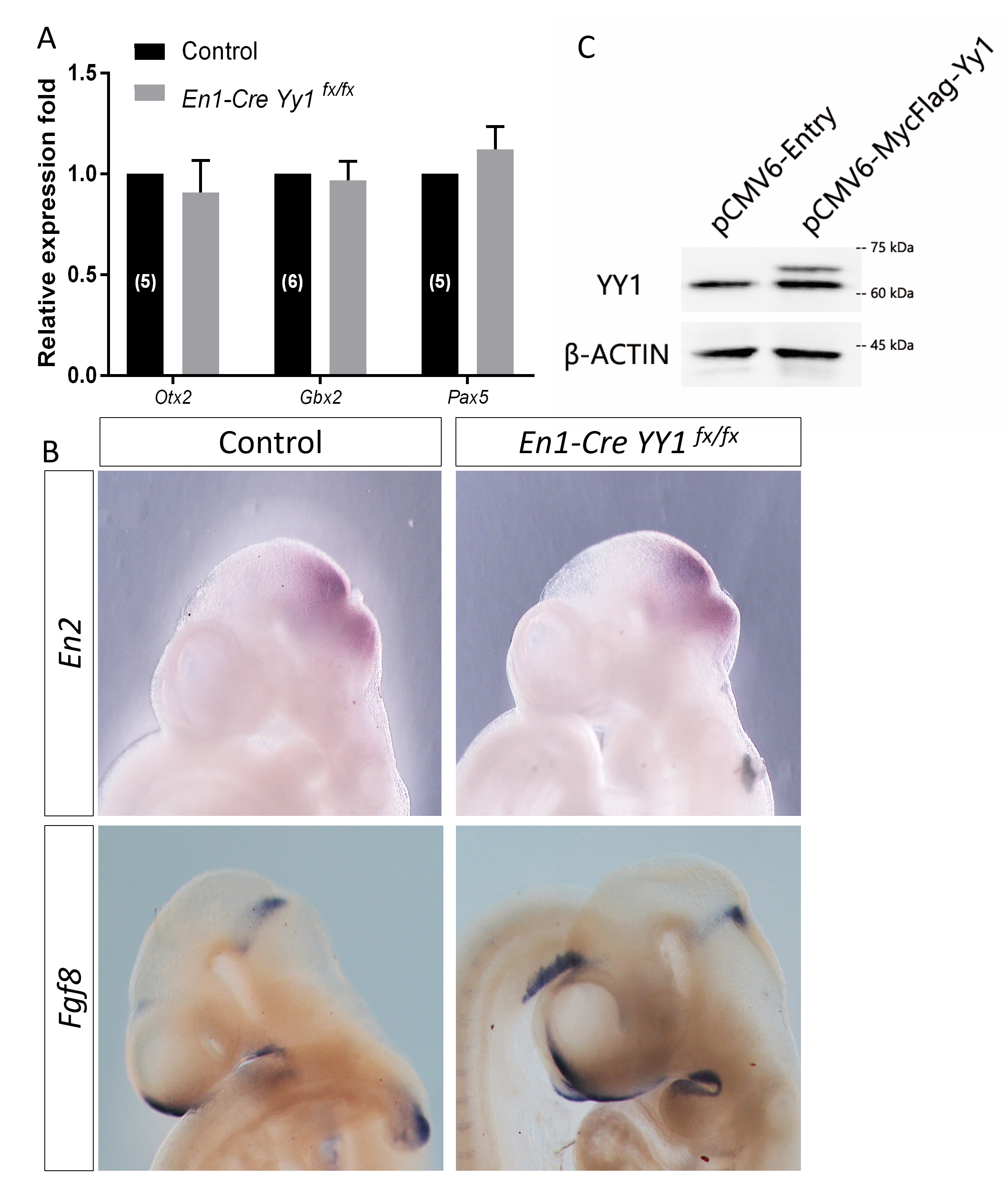

Supplement: Supplementary file 3 — Additional file 3: Figure S3. (A) Real-time qPCR results showing no significant change of the regionalization markers Otx2, Gbx2 and Pax5 in E9.5 mutant mes/r1 region. (B) Whole-mount RNA in situ hybridization figures showing normal expression of En2 and Fgf8 in the Yy1-ablated mes/r1 region comparing to the control littermates. (C) Western blot analysis showing YY1 overexpression in C17.2 cells transfected with the pCMV6-MycFlag-Yy1 plasmid. [file 13041_2020_643_MOESM3_ESM.tif]
